# On Medical Progress in Bristol
*Based on the Long Fox Memorial Lecture, delivered in the University of Bristol on 7th November 1963. A more complete version of this Lecture, including the illustrations used, has been published in book form and is obtainable from John Wright & Sons Ltd., The Triangle, Bristol. Price 12/6.


**Published:** 1964-10

**Authors:** A. V. Neale

**Affiliations:** Professor of Child Health, University of Bristol


### ON MEDICAL PROGRESS IN BRISTOL*

BY

A. V. NEALE, M.D., F.R.C.P.

*Professor of Child Health, University of Bristol*

Edward Long Fox was born in 1832. About a hundred years previously the Bristol Infirmary was founded, it being recorded that "persons of all persuasions" took part in the ceremony and that the motto, "Charity Universal" truly represented the broad-minded catholic spirit of both churchmen and dissenters who united in working for the new charity.

John Bonython (1695–1761), the first physician, was a good start—he being tender-hearted, kind, helpful to the poor and needy, exceptionally well-educated and having the grace of a gentleman. Six months later the first Matron, Mrs. Ann Hughes, commenced her work (at £15 p.a.) and continued, with great ability and integrity, for 39 years (d. 1770). The House Visitors admitted and dismissed the patients on Mondays and Thursdays. Rules were read in the wards every Tuesday morning. Mothers and near-relatives of patients were allowed to help nurse their friends. Card playing and dice games were not allowed and smoking was forbidden anywhere within doors. No patients were allowed to lie in bed with their clothes on. The Matron was the Governor during the absence of the House Visitors and Staff; it was her duty to see that all inmates returned to their ward betimes and in a sober condition. Political discussions were current on the merits of George the Second, but the Staff of the Infirmary were nearly all for Bonnie Prince Charles. This was to the accompaniment of Dr. Bonython in his laced ruffles, large wig, red cloak, long-frocked coat, gold-headed cane, or one of the surgeons (Mr. Wm. Thornbill) in his black velvet suit and French rapier. Even the student-apprentices went about with red cloaks and swords. Staff lived near the Infirmary, not in the neighbouring villages of Clifton or Redland for night visits were risky; highway robbery was not rare. Meat was cheaper than rice (good ox-beef 1½d. per lb.) and was liberal in the diet, but it is notable that in 1762 about 17,000 gallons of ale or beer were used each year and about 5,000 gallons of milk (at 4d. a gallon in the summer).

Pressure on beds was great—there were 50 beds (increased to 130 in 1755). Beds were placed in the passage and in case of urgent need two patients were put into one bed. The Visitors' Committee closely questioned every patient and rigidly refused any who could, apparently, afford to pay for medical attendance. Admission of patients with ulcerated eyes was strictly limited because there was only one cold bath and one warm bathing tub. There was an order for keeping the windows shut, but later the Quarterly Board appealed to the medical staff to encourage a ventilator system. They did so reluctantly, believing that night air and draughts caused diseases.

The surgeons operated on ordinary wooden tables; brandy and laudanum were the only narcotics. The average number of people bled at the Infirmary was 32 per day; one pupil bled 560 patients at an average of 12 ozs. The surgeons gained reputation in "cutting" for the stone which was duly rewarded by special notice in the daily newspaper. Medical diagnoses were nebulous, "low spirits", hypochondria, "impostumations" or inflammatory swellings, but hydrophobia and its horrors were not rare in Bristol. Notably, Dr. Wm. Cadogan of Clifton was tackling the many dangerous diseases in children and taught on the care of infants; his famous treatise appeared in 1748 and was full of valuable information, new ideas and methods.

* Based on the Long Fox Memorial Lecture, delivered in the University of Bristol on 7th November 1963. A more complete version of this Lecture, including the illustrations used, has been published in book form and is obtainable from John Wright & Sons Ltd., The Triangle, Bristol. Price 12/6.

Medical affairs around Bristol were lively—Edward Jenner (1795) pursuing the prevention of smallpox, Caleb Parry (1786) writing originally on exophthalmic goitre (at Bath), Robert Malthus producing his famous essay on the Principle of Population (1798), Humphry Davy (1799) establishing the anaesthetic values of nitrous oxide. Davy (1800) wrote his immortal paragraph—"As nitrous oxide appears very capable of destroying pain, it may probably be used with advantage during surgical operations"; and Robert Southey thought that "the atmosphere of the highest of all possible heavens must be composed of this wonderful gas". Unhappily, many more patients were to suffer pain before Davy's great discovery was put to common use.

Dr. Richard Smith (senior) founded the Infirmary Museum about 1775—it was the best of its type in England at that time and it was further extended by Richard Smith (junior). Great interest was taken in this museum and many surgeons from London visited it. The specimens, many of special forensic interest, were used for teaching the student-apprentices. Body-snatching was still frequent in the cemetery near the Infirmary. Also, the bodies of executed criminals were brought into the Committee Room and "there lectured on". However, with the founding of the Bristol General Hospital in 1831 these gruesome events seemed to diminish. In fact, it was advocated that the General Hospital would prove to have "a rational appeal to humanity and benevolence". Rivalry between the two institutions was to prove a stimulus to both. A long line of brilliant progress had commenced whilst Edward Long Fox was in his infancy.

This was the time when the Foxes entered into the history of the Infirmary. E. L. Fox, senior (Physician 1786–1816), showed an early insight into the problems of mental illness and adopted humane methods and believed in the "non-restraint" system, probably the earliest to do so. He was called in consultation by King George III. Fox, senior, attributed cholera to a "living animalcular virus". He was a firm believer in bacterial aetiology long before Louis Pasteur or Lord Lister. His "busy, useful and thoughtful life" was a good omen for his future distinguished grandson who was born (1832) 3 years before his grandfather died (1835).

### EDWARD LONG FOX

During Edward Long Fox's student days (at Shrewsbury School, Balliol College, Edinburgh and London), the Infirmary became "Royal" (1850) and seven years later, at the early age of 25 years, Fox was appointed to its staff as Physician.

Nurtured at Balliol College, with a First in Natural Science Tripos (1855), clinical clerk to Bence-Jones, house physician to Charles West, and to Sir Andrew Clarke, Fox started under good influence. He had, as John Beddoe put it: "a mind that was clear, healthy and serviceable; and zeal and industry of good example". A little conscious of his early appointment, he said to his ward students "I wish to say that as I have only recently passed the student stage myself I shall feel pleased, should anyone of you notice anything overlooked in my ward walk and practice that might be of importance in the care and treatment of the patients, if you would kindly remind me of the fact". His "logical alertness, sure and unerring judgment and optimism in prognosis" apparently gave the students little hope of finding any criticism. His senior physician was William Budd who had brought a splendid intellect and an intense love of his calling to his work among his patients. Budd had been elected F.R.S. in 1850, and in 1856 had pronounced upon typhoid fever, declaring it to arise from contagion from the diarrhoeal discharges, defects in sewers, and contamination of wells.

Fox had met a master-mind. Budd and Fox must have had many discussions together about typhoid and about tuberculosis. Budd's book on Typhoid Fever (1873)

must have attracted the deep interest of Fox, if only for its magnificent coloured drawings of the intestinal lesions. All must agree that it is a great medical classic. What must Fox have thought when he opened *The Lancet* (1867, *2*, 451) to find a letter by Budd which argued that tuberculosis is a "true zymotic disease of a specific nature in the same sense as typhoid, scarlet fever, or syphilis, and has a law of contagious succession, the tuberculous matter itself does include the specific morbific material by which phthisis is propagated from one person to another and so disseminates throughout society?" And all this fifteen years before Robert Koch discovered the tubercle bacillus!

Fox was, quite early, attracted to problems concerning the nervous system. Of representative type, we should look at his accurate neurological descriptions of two cases of spinal cord compression due to "encapsulated tumour" (probably meningioma); each patient died a lingering death from gradually progressive paraplegia. He had accurately localized the tumours on the basis of meticulous clinical signs, but, of course, he sadly deplored the absence of appropriate surgery at the time. He must have been thoughtful on what he could possibly do for congenital meningocoele. He photographed an example with spina bifida ($D_8$—$L_1$) in which spontaneous closure of the meningocoele occurred but hydrocephalus followed. He correctly argued that there was a dual relationship in arrested development.

Chorea Sancti Viti (then common) caused Fox to speculate on the variability of "medical constitutions"; he attempted to probe the possible causes—fear, worry, family "neurosis", anxiety, and the "rheumatic diathesis" as exciting causes. He encouraged improved nutrition, quietness and skilled nursing, but had no use for the vast number of empirical and strict ritual treatments then in vogue.

Fox was versatile—on enlargements of the spleen he applied his mind to a physiological-pathological study in which he fully grasped the mechanical effects of "incompetent liver" and "impeded portal circulation". The clinical significance of splenomegaly in miliary tuberculosis and in typhoid fever was emphasized. He regretted that no-one had discovered the cause of the splenomegaly in the "adénie of Trousseau" (or Hodgkin's disease) or of leucocythaemia (leukaemia)! We are still as ignorant as he was. As an angle on Fox's ideas, I mention his enthusiasm for "Mangana Water" from Cransac, S. France; he believed in the benefit of the "trace" elements, particularly manganese, in chronic intestinal diseases (with presumably malabsorption). He was probably right.

Dr. Fox's first paper, on "Traumatic Tetanus" appeared in 1860, and his last one, on "The Physical Advantages of Abstinence", in 1898. Fox entered skilfully and kindly into group discussion. A good example was that on Intestinal Obstruction (1885). Greig Smith opened, against the views of some—"rest, starvation and opium, rectal feeding and much belladonna". Smith pressed that where diagnosis was uncertain and the symptoms acute, one should operate at once. Fox said: "most cases of intestinal obstruction could be relieved by the surgeon if only the physician had not touched the case previously". Absurd argument was to be avoided. "It is an evidence of a more sensible feeling, when surgeons, with the courage now induced by the use of antiseptics, meet physicians, who have acted on the grand principle of physiological rest, in friendly conference on the question of obstructions of the bowel".

Fox's work on the "Influence of the Sympathetic" (1882) was more than ordinary. He had probably remembered Wm. Harvey's words on the influence of emotions "every affection of the mind, pain, hope or fear, is the cause of an agitation whose influence even extended to the heart" (De motu cordis, 1628). Fox's *magnum opus*—"The Pathological Anatomy of the Nervous Centres" (1874) was a valiant attempt to understand and describe inflammations, degenerations and neoplasms in the cerebrospinal system. Much of his writing links with that of other great neurologists of his own time—Hughlings Jackson, J. M. Charcot, William Gowers. Fox pictured disease in the nervous system as nature's most delicate experiment, the secrets of which he hoped might be better elucidated by improved micro-pathological methods. As early as 1866 he studied aphasia in its association with right hemiplegia; he postulated a "localization for articulate speech in the posterior part of the left frontal convolution of the brain". He carefully noted that in cases of left hemiplegia, speech was usually not lost.

After Wm. Budd's death (1880) Fox maintained a searching interest in tuberculosis. He was very active in the National Association for the Prevention of Consumption. Other epidemic diseases caused him alarm. Diphtheria was rampant; of 541 cases in Bristol (1894–1896) 122 died. In 1863 the people of Bristol had been grateful to Fox for his sense and energy in controlling the typhus epidemic which struck the "over-crowded poorer parts of Bristol" in that year. Significantly, a great cholera epidemic struck Bristol in 1832 (the year of Fox's birth and the opening of the B.G.H.) and the next severe cholera outbreak commenced just after he was appointed Physician at B.R.I., an epidemic which he faced with great courage and in which Bristol placed complete faith in him to take control, which he gladly did with eminent success and thus rightly deserved the early general popularity which came to him.

But he had his troubles, for he told Beddoe that "there is nothing to bring on a fit of gout like a run of anxious cases". However, despite this disability he had equanimity; he did much to maintain a "healthy social and ethical tone in the whole professional body" in Bristol. In fact, he had "love, honour and troops of friends" who later took the necessary steps to establish this Lecture in his memory. Carey Coombs's father knew Fox and was struck by "the student spirit within him—a spirit of ceaseless enquiry". Fox was a practical man and his interests in prevention of phthisis caused him to be a leader in the founding of Winsley Sanatorium. Vision, purpose and dedication were his working tools.

Fox expounded "in temperate and thoughtful terms" the relationship which medicine should bear to the State. There should, he said, be emphasis on close co-operation between the natural, the applied, and the social sciences. In this he hoped for "a good medical library and a large convenient room for professional meetings in the University College". He would certainly be happy to see the new and recent developments in the Medical School, the growth and vigour and its present library of 43,400 volumes (1962), and he would be a little astonished to know that the present (1963) membership of the Bristol Medico-Chirurgical Society is 517.

### LONG FOX'S CONTEMPORARIES

At about the same time James Greig Smith was energetically overhauling the surgical sphere in Bristol. All were grateful for his "impetuous energy" in getting things done and wiping out "all well-meant superfluities which would never do". Like many tired over-worked valuable men of his time, Greig Smith died from pneumonia at the early age of 43 (1897). His Aberdeen accent had bothered some and confused many. He gave great impulse to the progress of surgery, particularly through his careful studies of surgical pathology. This excellent surgical work was to be followed up by James Paul Bush, George Munro Smith, James Swain and Thomas Carwardine.

Surgical operations were becoming more successful; the results of earlier diagnosis were improving. A. W. Prichard (B.R.I.), and James Swain (Surgeon, B.R.I.) were happier in the increasing surgical cures of acute appendicitis (1894). J. Swain (1897) pursued the early clinical use of Röntgen rays: he studied the lamination of stones in the renal tract, their related chemistry and causes. In the same year, J. Michell Clarke (Physician, B.G.H.) was looking at new ways of treating some of the "severe anaemias". He tried the inhalation of oxygen, and the effects of transfusing 6 ozs. of "normal venous blood" in pernicious anaemia. He was troubled by the occurrence of haemoglobinuria with renal casts in some cases after the transfusion. This was six years before Karl Landsteiner discovered the blood groups.

Appendicitis was a great problem. Although the American surgeon, Charles McBurney, received credit for the diagnostic "point" of tenderness in 1889, it should be recalled that the records of the B.R.I. of 1834 indicate that Mr. Frederick Granger of Bristol described clearly and accurately this diagnostic sign in acute appendicitis—it should, in fact, be remembered as "Granger's point".

G. Munro Smith (when Demonstrator in Physiology, 1883) began to follow the "cardiograph" in medicine—"this arteriography helps to amuse restless medical students as a scientific toy"—but, he said, "the physiology of to-day is the pathology of tomorrow especially in the domain of heart disease"; he impressed his listeners that "cardiograms" might become a fruitful field of research in clinical work. A lasting truth indeed!

The day-to-day work was clinically exciting. W. H. Harcourt (B.R.I.) noted a family which attended surgical out-patients—seven children with congenital syphilis, mother with gummatous ulcers and the father with chronic syphilitic osteitis. There were many cases of lupus vulgaris of extreme severity. Epidemics of tetanus were shocking, some from operations and some from street wounds. Dr. A. Sheen confessed that tetanus was a "complete mystery" but he was fully aware that "as long as the slightest evidence of disease exists a sudden spasm of the glottis may at any time destroy life".

At this time (1883) A. B. Prowse (Physician, B.R.I.) introduced retinoscopy as a means of diagnosing and estimating ocular errors of refraction, of special value for children with myopia in which there is a real need for accurately adapted eyeglasses as early as possible. This was a fine development. The first Eye Dispensary in Bristol had opened in 1812.

The Bristol General Hospital opened its wards in 1832 (the year of Edward Long Fox's birth) and in 1854 the Maternity Department in Bristol started in the B.G.H., Joseph G. Swayne being the Physician-Accoucheur. His early reports were ominous. Of 22 cases of placenta praevia there were 8 maternal deaths and 17 perinatal deaths. No wonder Swayne was worried and distressed about "the losses of blood which occur suddenly and in the absence of professional assistance". At the same time, Dr. J. Beddoe, F.R.S., speaking in the Medical School, said: "the physician may be more akin to the philosopher or a student of natural history, but the extent and complexity of human knowledge is so great that no man can hope to approach its boundaries except at a few points". However, he said that "wide culture need not detract from practice ability, but narrowness is by no means always correlated with intensity". Much was to be extracted from these wise remarks in the future developments of safer midwifery in Bristol. Swayne diligently continued his clinical researches and methods. In 1891 he pressed for more knowledge on "eclampsia" in which there was 50 per cent foetal-neonatal mortality. The eclamptic syndome occurred in 1 in 80 single pregnancies and in 1 in 12 twin pregnancies, and 66 per cent were primiparae. Swayne struggled to give all aid even if heroic—he advised blood-letting to 30 ozs. "in a full stream from a large vein, leeching, and cold packs to the head". Unfortunately, in one series 11 out of 36 mothers died. Five years later (1896) Swayne (now Emeritus Professor of Midwifery) had started "Induced Premature Labour" for conditions not obstructing labour; this was mainly to prevent eclampsia in "toxaemia" of pregnancy. Prevention of obstetric troubles was, however, difficult. A. E. A. Lawrence (Professor of Midwifery and Swayne's successor at B.G.H.) said "no woman ought to marry if she has an ovarian or a broad ligament cyst or a uterine fibroid; these should be all remedied before marriage as they should not be allowed to interfere with the due performance of the marriage contract".

The "white man's plague" (phthisis) was rampant in Bristol. Shingleton Smith persevered in the attack upon it; in 1888 he even tried subcutaneous and intrapulmonary injections of carbolate of camphor. Pressing further on with the "germ theory" and its special relationship to phthisis, Smith pursued treatments with inhalations of pulverized biniodide of mercury, but wisely said this must be supported by combatting anorexia; that the battle in the germ theory turned on the "nutritive processes of the body and the tissue protoplasm against the tubercle bacilli". This was sound sense.

We must give record at this point to Theodore Fisher, one-time Pathologist, B.R.I., and Physician, Children's Hospital (1896), for his model studies of morbid anatomy; his most important contributions concerned the serious effects of rheumatic carditis and tuberculous pericarditis (1895).

At the B.R.I. Fisher was obviously confronted with many deaths due to "fibroid degeneration of the myocardium" (old infarcts) and he argued against a rheumatic cause and took special notice of the atheromatous state of the coronary arteries. It is noteworthy that Edward Jenner (1772) (who was invited to go with Captain Cook on his second expedition round the world but who preferred to be a medical apprentice to John Hunter), when aged only 22 years, observed the clinical relationship of angina pectoris with heart disease; at autopsy he was so surprised when his knife struck against something hard and gritty in the heart that he looked up at the ceiling thinking some plaster had fallen down! Jenner's paper (*c.* 1800) "Remarks on a Disease of the Heart following acute Rheumatism" antedated all others on this subject by twenty years. Fisher stressed the special significance of myocarditis in rheumatic disease of the heart at a time when emphasis was almost entirely on the valvular lesions (1900). This was five years before L. Aschoff described the special histological nature of the myocarditis in acute rheumatism in 1905. Two other matters showed Fisher's care and ability. First he described occlusion of the hepatic vein (now the so-called Hans Chiari syndrome) near its junction with the inferior vena cava and its consequential effects in the liver, noted absence of enlarged spleen, and that no liver cirrhosis occurred but that ascites did occur. His drawing of the adaptive venous anastomosis reveals his mastery of pathology. It may be that this was the earliest description of this condition. Secondly, he studied hypertrophic pyloric stenosis in infants. He observed that on the day before death the visible gastric peristalsis ceases and the stomach became very distended and dilated. We now know the reason—alkalosis, hypokalaemia and dehydration. Conrad Ramstedt's first operation for pyloric stenosis was done nine years later.

### CAREY COOMBS

Whilst a demonstrator in pathology in the University College, Bristol (1907), and physician at the Children's Hospital, Carey F. Coombs wrote his first paper on Rheumatic Carditis in Childhood, an appropriate follow-up of Theodore Fisher's work seven years earlier. Coombs noted 60 per cent of children with acute rheumatism had signs of carditis. He said "it is carditis and the rheumatic infection is blood borne"; that the gravity in the childhood affection lay mainly in the myocarditis and that it was "remarkably apt to recur". He remarked on mid-diastolic murmurs in the mitral area (in early rheumatic carditis particularly) this murmur being possibly related to effects of the enlarged or dilated ventricle (emphasis again on the myocarditis), and this was compared with the pre-systolic murmur of mitral stenosis where auricular systole played a more important part. Coombs hoped that the work which he and his local colleagues were doing in Bristol would bring order into some of the desperate chaos concerning heart disease. In 1914 he commenced cardioscopy, using a fluorescent screen, thus demonstrating the enlarged left auricle in mitral stenosis; the "bismuth swallow being diverted to the right and there being some oesophageal delay at that level".

C. Coombs and Geoffrey Hadfield probed the mysteries of streptococci, including the "natural history of ulcerative endocarditis". They asked —"why should these streptococci be so specifically pathogenic and why should they cause sometimes one kind of heart lesion (acute carditis) and at another ulcerative endocarditis?". Could it be presumed that in acute rheumatism the infection arose from one single dose, whereas the ulcerative disease could be due to recurrent massive doses in the circulation? Why are bacteria arrested on the irregular surface of an abnormal or diseased heart valve? From 1927 a University Centre for Cardiac Research got into action; in 1931 work had accumulated in field research regarding conditions favouring occurrence of rheumatic heart disease in children. The pathology of the rheumatic lesions, of ulcerative endocarditis, staphylococcal pericarditis, syphilis of the cardiovascular system, thyrotoxic cardiopathy, hypertensive disease, coronary sclerosis, and spinal or thoracic deformities and their effects on the heart were studied. Congenital heart disease came in for careful observation; during medical inspection of school-children, 116 were found to have undoubted congenital morbis cordis. Coombs and his team knew that this had "importance beyond that of the mechanical consequences", there was always the basis of the (then) fatal ulcerative endocarditis. In view of the rapid growth of all this research, Coombs pleaded for provision for research in Medicine; he hoped that eventually a University-Hospital Centre for Research would be established. F. J. Poynton, Coombs's colleague and friend, painted a dismal picture. "The most deadly form of rheumatic heart disease is the apyrexial type, with a stealthy carditis, nodules and persistent anaemia—there was no real remedy, and even splendid nursing often failed to give recovery." But, in Coombs's final words (he died in 1933) he made the prophetic reply—"that rheumatic carditis does recover sometimes, and one day cardiac rheumatism will be prevented.

### MENTAL HEALTH

In Bristol the first hospital for diseases of the mind was opened by John Carey in 1697. Melancholia, said F. H. Edgeworth (B.R.I. 1898), is a disease that any human being who has reached the battle of life can enter into, but depression "is not necessarily melancholia". Michell Clarke (1898) considered "giant urticaria a nervous affection"; it could be produced by hypnosis. J. Paul Bush (Surgeon, B.R.I.) thought there was a short cut in the cure of mental illness. Three cases nearly convinced him. A young woman who had been "under restraint for an unhinged mind for 3 years" had an operation for severe ingrowing toe nails, this was followed by "mental recovery". A maniacal woman aged 46 years "recovered" after drainage of subphrenic abscess. A melancholic woman aged 32 years was "quite well" 3 months after the removal of numerous pins and other ingested articles from the stomach! Even incisions apparently have psychotherapeutic influences, at least so some of Bush's colleagues thought in 1898.

In 1922 an opinion was given that every medical student should be instructed in the rudiments of psychology in conjunction with biology and physiology—"Bristol being pre-eminently fitted for such reform as an expression of the erudition of Professor C. Lloyd Morgan". H. H. Carleton (1920) differentiated hysteria from malingering. He also expressed the urgent need for early accurate diagnosis and treatment of all forms of insanity. Carleton considered the relationships of "Habit and Illness," the elucidation of significance of symptoms, of psychosomatic troubles, and true problems of the "professional invalid".

E. Long Fox (in his Presidential Address, B. M. A. in Bristol, 1894) commented on the attitudes of the time to possible mental illness or mental retardation. He said "the mentally feeble and the criminal subjects" should be looked into by a scientific enquiry. He hoped for increasing possibilities in the care and treatment of physically and mentally feeble children, if only by teaching the object lessons alone. He referred to the possible medical influences in social life and especially in the sensible care of children; he commented that the "troubles of the railway pace of life" were aiding crime and insecurity! Stresses and strains there were, no doubt, even in those Victorian days.

### POSTGRADUATE MEDICAL EDUCATION

Thoughts on medical post-graduate education were raised soon after the University appeared (1909). Dr. J. M. Rattey (a senior general practitioner at Frome) said we should reverse the undergraduate attitude of "cramming much and thinking little" and consider more the "whys and wherefors" of things. He hoped that the "abundant store of material in the University" would become available to the medical practitioners of the West of England for postgraduate tuition, especially for those who realize that "what was taught 40 years ago is much out of date", but also for those who wish to study for higher degrees or diplomas. Ten years later (1919) Dr. J. M. Fortescue-Brickdale (B.R.I.) deplored the very slow advance made towards post-graduate education. He suggested that the State should endow post-graduate medical education "which must in the end benefit the whole population". Another general practitioner—Dr. C. E. S. Fleming, Bradford-on-Avon—raised the subject again in 1932, particularly in regard to "the unsatisfactory state of midwifery and the defective medical education" which had led "some doctors to have such ignorance and incapacity which is only cloaked by dogmatism and craft". Fleming rightly said that "no system of medical education is anywhere near complete without ample provision for post-graduate study". To-day we are in no doubt whatever about this. In fact, the Minister of Health (1963) has set aside £250,000 per annum to be known as the Post-Graduate Education Fund for general medical practitioners, and a generous donation has been given by the Nuffield Foundation for general development of the medical post-graduate work of the Universities and Regional Boards.

On 29th May 1902, at a meeting at the Chapter House of Bristol Cathedral, it was announced that there would be "an annual lecture at University College on some subject connected with medical science". The Long Fox Memorial Lectures have given opportunity for some of our predecessors to express their special ideas.

Fox was a true follower of William Harvey (1578–1657) in the scientific attitude and in the need to understand ourselves and others to create friendship. He appreciated that they also serve who only stand and think, and that they serve more who generate kindness and possess the art of communication and helpful guidance. He was aware that the purpose of education is understanding.

The last words recorded of the man in whose memory this Lecture is given were: "The sands of time are sinking year by year for all of us, but the memory of the great ones in the past is a spur to further effort, an example for a truer and higher life in us all. Happy shall we be if we can pass to our successors some of the professional honour and scientific attainment which has been handed down to us from our predecessors".

